# *HNF1A* regulates colorectal cancer progression and drug resistance as a downstream of *POU5F1*

**DOI:** 10.1038/s41598-021-89126-2

**Published:** 2021-05-14

**Authors:** Shiki Fujino, Norikatsu Miyoshi, Aya Ito, Masayoshi Yasui, Chu Matsuda, Masayuki Ohue, Mamoru Uemura, Tsunekazu Mizushima, Yuichiro Doki, Hidetoshi Eguchi

**Affiliations:** 1 Department of Gastroenterological Surgery, Osaka University Graduate School of Medicine, 2-2, Yamadaoka, Suita-City, Osaka 565-0871 Japan; 2 Innovative Oncology Research and Translational Medicine, Osaka International Cancer Institute, 3-1-69, Otemae, Chuo-ku, Osaka, 541-8567 Japan; 3 Department of Surgery, Osaka International Cancer Institute, 3-1-69, Otemae, Chuo-ku, Osaka, 541-8567 Japan

**Keywords:** Cancer, Cell biology

## Abstract

POU5F1-expressing cells can self-renew and differentiate, contributing to metastasis formation in colorectal cancer (CRC), but it plays an important role in normal pluripotent stem cells. Here, we identified the CRC-specific gene, HNF1A, which is the downstream of POU5F1. HNF1A associates with fatty acid and glucose metabolism, and CRC cells highly expressed it. In 198 CRC patients, high HNF1A expression was an independent predictor of disease-free (*P* = 0.031) and overall (*P* = 0.007) survival. HNF1A-knockdown showed significantly reduced cell growth, increased apoptosis, and improved anticancer drug sensitivity. We revealed that HNF1A regulated controlled GLUT1 expression via HIF1A and multidrug resistance protein function to suppress SRI. HNF1A expression was elevated in persister cells after exposure to anticancer drugs, and anticancer drug sensitivity was also improved in persister cells via the inhibition of HNF1A. In conclusion, HNF1A expression can reflect resistance to anticancer drug treatment, and its suppression improves anticancer drug sensitivity as a new therapeutic target.

## Introduction

The number of patients with cancer has increased, with approximately 17.2 million patients and approximately 8.9 million cancer-related deaths worldwide in 2016^1^. Colorectal cancer (CRC) is the second leading type of cancer and the third leading cause of cancer-related deaths globally^1^. CRC is also the second leading cause of cancer-associated death in Japan and leading cause of cancer-related death among women in Japan in 2017^2^. For CRC, complete surgical tumor resection including removal of the primary tumor, regional lymph node, and distant lesions such as liver and lung metastasis is effective^3,4^. However, recurrence occurs in 17.3% of patients treated by complete resection^5^. Despite recent advancements for treating patients with metastatic CRC (mCRC)^4^, the 5-year survival rate of is only 18.8% for stage IV patients with distant mCRC^5^. Thus, a more effective approach for treating distant metastasis is necessary to improve the prognosis of patients with mCRC.

We previously reported that high *POU5F1* expression is an independent prognostic factor for distant metastasis in CRC^6^ and promotes liver metastasis^7^. However, *POU5F1* is also expressed in normal tissue stem cells and is involved in cell proliferation and differentiation^8,9^. There are not any anti-cancer drugs targeting POU5F1, and directly targeting POU5F1 seems to be difficult in CRC treatment. Thus, in this study, we focused on genes downstream of *POU5F1* and highly expresses in CRC cells as therapeutic targets.

Hepatocyte nuclear factor 1A *(HNF1A)* is located on chromosome 12 and encodes a transcription factor, belonging to the POU domain protein family that is required for the expression of several liver-specific genes^10,11^. *HNF1A* mutation is well-known to be associated with fatty acid and glucose metabolism^12,13^, and caused maturity-onset diabetes of the young, type 3^14^ and coronary artery diseases^13^.

In CRC, frequent mutations in *HNF1A* were reported in microsatellite instability-high (MSI-H) tumors^15^, but mutation rates in all colorectal cancer patients, including microsatellite stable (MSS) tumors were only 2%^16^. A study of two independent cohorts of patients with mCRC treated with irinotecan-based chemotherapy showed that patients with the HNF1A-coding variant p.I27L have a good prognosis compared to those without the variant^17^. However, the significance of HNF1A expression and its role in colorectal cancer are mostly unknown. In this study, we revealed the relationship between *POU5F1* and *HNF1A* and focused on *HNF1A* expression as a key determinant of chemotherapy success.

## Results

### Search for genes located downstream of POU5F1 in clinical colorectal cancer

**Figure 1:**
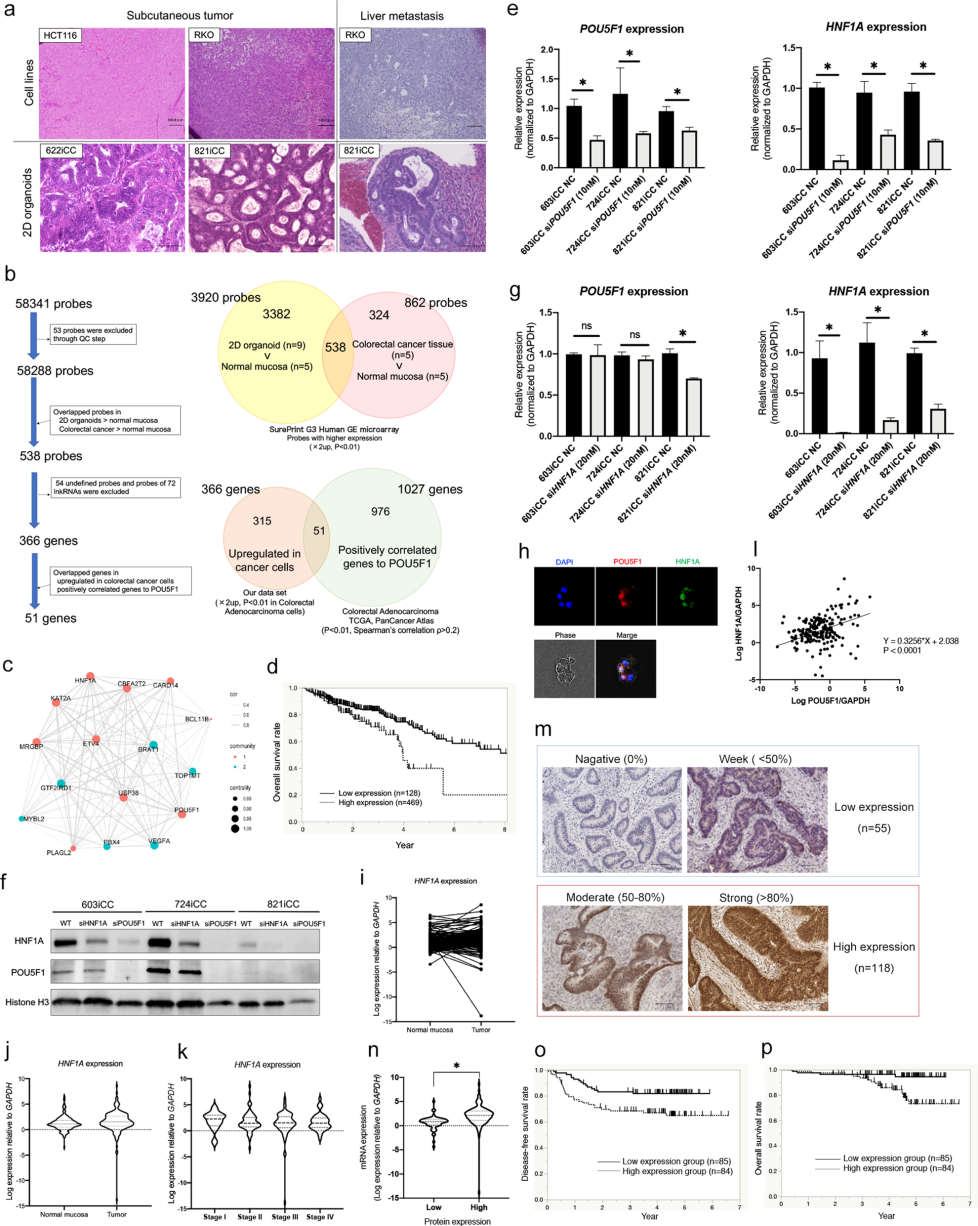
HNF1A as a downstream of POU5F1 and its expression in clinical tissue specimens and prognosis. (**a**) H&E staining of xenograft tumors from cell lines (HCT116 and RKO) and primary cultured 2D organoids (2DOs) (622iCC and 821iCC). Histological examination of 2DO showed tubular adenocarcinoma in both subcutaneous tumors and liver metastasis. (**b**) Chart of selection of genes related to POU5F1 as a therapeutic target. A total of 366 genes (410 probes) were identified, which were higher in 2DOs than in normal mucosa, and higher in colorectal cancer than in normal mucosa (more than twofold, *P* < 0.01). Positively correlated 1027 genes to POU5F1 were selected from TCGA database (*P* < 0.01, Spearman’s correlation ρ > 0.2). Fifty-one genes overlapped in these data sets. (**c**) Gene network of extracted 16 genes was visualized based on a random graph of 16 nodes and a wiring probability of 0.3 resulting in 120 edges between nodes. Nodes are sized by degree of centrality and colored by community class. (**d**) Overall survival (OS) curves based on *HNF1A* mRNA expression from TCGA database. Patients with high *HNF1A* expression levels had a poor prognosis compared with low *HNF1A* expression (*P* = 0.004, log-rank test). (**e**) The reductions in *POU5F1* and *HNF1A* expression were significant with *POU5F1* siRNA (si*POU5F1*) compared to levels in the siRNA negative control (NC) groups in all three 2DOs (n = 4). Values are presented as means ± SEM (*P < 0.05, Wilcoxon’s rank sum test). (**f**) Western blotting of HNF1A and POU5F1 in siPOU5F1 and si HNF1A three 2DOs. (**g**) The reductions in *HNF1A* expression were significant with *HNF1A* siRNA (si*HNF1A*) compared to levels in the siRNA negative control (NC) groups in all three 2DOs. The reductions in *POU5F1* expression were not significant in 2 2DOs (n = 4). Values are presented as the means ± SEM (*P < 0.05, Wilcoxon’s rank sum test). (**h**) Immunocytochemistry of HNF1A and POU5F1 in cultured 2DO (603iCC). (**i**–**l**) *HNF1A* mRNA expression in 198 clinical tissue specimens. (**i**) *HNF1A* expression normalized to *GAPDH* expression in 198 tumor tissue and corresponding normal tissues was shown. (**j**) *HNF1A* expression in tumor tissues (median, 2.89) was higher than in the corresponding normal tissues (median, 2.08) (*P* = 0.001, Wilcoxon’s rank sum test). (**k**) *HNF1A* mRNA expression in tumor tissues was not correlated with TNM stage (Tukey’s test). (**l**) The correlation between *HNF1A* expression and *POU5F1*expression in tumor tissue. (**m**) HNF1A protein expression in clinical tissue specimens. HNF1A staining was mostly in nuclear. According to the staining area, 55 samples were classified into the low expression group and 118 were into the high expression group. Scale bars, 100 μm. (**n**) The correlation of HNF1A expression between *mRNA e*xpression and protein expression in tumor tissue (*P < 0.05, Wilcoxon’s rank sum test). (**o**) Disease-free survival (DFS) curves based on *HNF1A* mRNA expression. Five-year DFS rates in patients with high and low *HNF1A* expression levels were 65% and 82%, respectively (*P* = 0.014, log-rank test). (**p**) Overall survival (OS) curves based on *HNF1A* mRNA expression. Five-year OS rates in patients with high and low *HNF1A* expression levels were 73% and 95%, respectively (*P* = 0.004, log-rank test).

Two-dimensional (2D) primary cultured cells named as 2D organoid (2DO) were established according to a previous report^18^. Nearly all cells of 2DO expressed epithelial cell adhesion molecule (EpCAM) like epithelial-derived tumor cells (Supplementary Figure S1). Therefore, compared with clinical tissues, 2DO was considered useful for searching gene expression specific to clinical cancer cells because cancer cells were specifically enriched. These cells showed more similar clinical tumor characteristics, such as ductal structure, in xenograft tumors created by subcutaneous transplantation and spleen injection compared with CRC cell lines (Fig. 1a). Using microarray data, the gene sets of nine 2DOs, five clinical CRC specimens, and five normal mucosae were compared to identify therapeutic target genes (Fig. 1b, Supplementary Figure S2). There were 538 overlapped probes which were significantly highly expressed in 2DOs and clinical CRCs. Probes with no gene assigned or linked RNAs were excluded, thus, we identified 366 genes. Next, 1027 significant genes positively correlated with *POU5F1* in clinical CRC tissue were identified using The Cancer Genome Atlas (TCGA) database^16^. Of the 366 genes and 1027 genes, overlapped 51 genes were listed in Supplementary Table S1. Furthermore, we performed gene ontology (GO) analysis of these 51 genes and *POU5F1* using DAVID^19,20^. Eleven enriched GO terms were identified (Supplementary Table S2), and 16 genes were extracted from tumor-related DO terms (Supplementary Table S3). The correlation of these gene expressions was examined by R (CRAN; the R Foundation for Statistical Computing, Vienna, Austria) using our microarray data sets mentioned above, and two communities were detected (Fig. 1c). Eight genes were classified into the same community as *POU5F1*. Additional analysis based on the Human Protein Atlas (Version: 18.1) using TCGA database^21,22^ showed that high expression of *HNF1A* significantly correlated with poor prognosis (*P* < 0.001; Fig. 1d, Supplementary Figure S3). Therefore, we focused on *HNF1A* as a gene associated with *POU5F1*. Next, the relationship between POU5F1 and HNF1A in clinical CRC was examined using 2DOs. Knockdown of *POU5F1* by short interfering RNA (siRNA) resulted in reduced HNF1A expression in 2DOs significantly (Fig. 1e,f, Supplementary Figure S4). Confirmation of knockdown of *POU5F1* was also made by suppressing *NANOG* expression (Supplementary Figure S5). However, knockdown of *HNF1A* did not reduce POU5F1 expression (Fig. 1g). Furthermore, immunocytochemistry of 2DO (603iCC) showed that HNF1A and POU5F1 co-localized in the nucleus of same cells (Fig. 1h). Thus, it was considered that POU5F1 regulated HNF1A and HNF1A was downstream of POU5F1 in CRC.

### High HNF1A expression correlated with poor prognosis in CRC

CRC specimens and adjacent normal mucosa of 198 patients were analyzed, and their characteristics are shown in Supplementary Table S4. *HNF1A* mRNA expression levels were determined by quantitative real-time reverse transcription polymerase chain reaction (RT-PCR), and were normalized as the *HNF1A/GAPDH* expression ratio for each sample. The expression of *HNF1A* in normal and cancerous parts of all patients is shown in Fig. 1i. The median HNF1A expression in the normal mucosa was 2.08 (range 0–89.38) and 2.89 (range 0–384.72) in tumor tissue, and it was higher in tumors than in normal mucosa (*P* = 0.001; Fig. 1j). It was not correlated with TNM stage (Fig. 1k). The expression of *HNF1A* was also positively correlated with *POU5F1* in these CRC specimens (*P* < 0.001; Fig. 1l). Next, HNF1A protein expression was compared to *HNF1A* mRNA expression in these primary CRC using immunohistochemistry (Fig. 1m). Twenty-five samples were inadequate for evaluation because of a lack of tissue, and 173 of the 198 patient samples were examined. All sections were examined independently and classified as follows according to the stained nuclear area. Negative for no staining, week for less than 50% staining, moderate for 50–80% staining, strong for 80% or more staining. Of these, negative and week were classified into the low HNF1A protein expression group and moderate and strong were classified into the high HNF1A protein expression group. The expression of mRNA in these two groups was shown in Fig. 1n, and *HNF1A* mRNA was correlated with protein levels in clinical CRC tissue (*P* < 0.001). Disease-free survival (DFS) and overall survival (OS) were examined in these patients. Four patients with multiple cancers and 25 patients with R1-2 resection were excluded, and finally 169 patients were analyzed. Patients were divided into two groups according to the median value of *HNF1A* expression. The relationships between clinicopathological factors and *HNF1A* expression status in the 169 patients are summarized in Table 1. *HNF1A* expression was not significantly correlated with any clinicopathological factors. Survival rates based on Kaplan–Meier analysis showed that patients in the high-*HNF1A* expression group had significantly lower DFS (*P* = 0.014) and OS (*P* = 0.004) rates than the low-expression group (Fig. 1o,p). The 5-year DFS rates for patients with high and low *HNF1A* expression were 65% and 82%, respectively, and the 5-year OS rates were 73% and 95%, respectively. According to univariate and multivariate analysis for DFS and OS, high *HNF1A* expression was a significant independent predictor of disease-free and overall survival in patients with CRC (Tables 2, 3). These results suggested that high expression of *HNF1A* plays affects in increasing malignancy in colorectal cancer. HNF1A protein expression was also examined in normal intestines, and HNF1A expression varied between cells (Supplementary Figure S6). Intestinal stem cells in crypt partially expressed HNF1A, but not all.

**Table 1:** Patient characteristics according to *HNF1A* mRNA expression.

Factors	Low expression group (n = 85)	High expression group (n = 84)	*P* value
Age (< 66/≥ 66)	42/43	44/40	0.700
Sex (male/female)	46/39	45/39	0.943
Histological grade (Others*/Tub1–2)	7/78	2/82	0.091
Tumor invasion (T3–4/T2)	78/7	75/9	0.582
Lymph node metastasis (N1–2/N0)	44/41	49/35	0.391
Lymphatic invasion (present/absent)	46/39	52/32	0.305
Vascular invasion (present/absent)	64/21	65/19	0.750

Tub1–2: well–moderately differentiated adenocarcinoma.

*Others: poorly differentiated, mucinous adenocarcinoma, or squamous cell carcinoma.

**Table 2:** Univariate and multivariate analyses of disease-free survival based on *HNF1A* expression and clinicopathological variables.

Factors	Univariate analysis			Multivariate analysis		
	HR	95% CI	*P* value	HR	95% CI	*P* value
Age (years) (< 66/≥ 66)	0.916	0.506–1.645	0.768			
Sex (male/female)	1.192	0.663–2.183	0.560			
Histological grade (Others*/Tub1–Tub2)	1.556	0.377–4.273	0.487			
Tumor invasion (T3–4/T2)	2.563	0.791–15.715	0.131			
Lymph node metastasis (N1–2/N0)	8.051	3.486–23.353	**< 0.001**	6.620	2.726–19.837	**< 0.001**
Lymphatic invasion (present/absent)	2.255	1.197–4.549	0.011	1.221	0.611–2.623	0.583
Vascular invasion (present/absent)	3.421	1.381–11.390	**0.006**	2.888	1.159–9.647	**0.020**
*HNF1A* expression (high/low)	2.154	1.167–4.138	**0.014**	1.979	1.063–3.831	**0.031**

*Others: poorly differentiated, mucinous adenocarcinoma, or squamous cell carcinoma.

The significance for the boldunderline indicate independent risk from multivariate analysis.

The significance for the underline indicate significant risk from univaliate analysis.

**Table 3:** Univariate and multivariate analyses of overall survival based on *HNF1A* expression and clinicopathological variables.

Factors	Univariate analysis			Multivariate analysis		
	HR	95% CI	*P* value	HR	95% CI	*P* value
Age (years) (< 66/≥ 66)	1.264	0.545–3.065	0.588			
Sex (male/female)	0.790	0.338–1.844	0.580			
Histological grade (Others*/Tub1–Tub2)	NA	NA	NA			
Tumor invasion (T3–4/T2)	2.837	0.591–50.906	0.231			
Lymph node metastasis (N1–2/N0)	5.804	1.976–24.711	**< 0.001**	4.573	1.530–19.636	**0.005**
Lymphatic invasion (present/absent)	1.887	0.775–5.263	0.185			
Vascular invasion (present/absent)	3.425	0.998–21.461	0.054			
*HNF1A* expression (high/low)	4.437	1.626–15.492	**0.003**	3.850	1.406–13.475	**0.007**

*Others: poorly differentiated, mucinous adenocarcinoma, or squamous cell carcinoma.

The significance for the boldunderline indicate independent risk from multivariate analysis.

### HNF1A inhibition reduced cell proliferation and improved drug sensitivity

**Figure 2:**
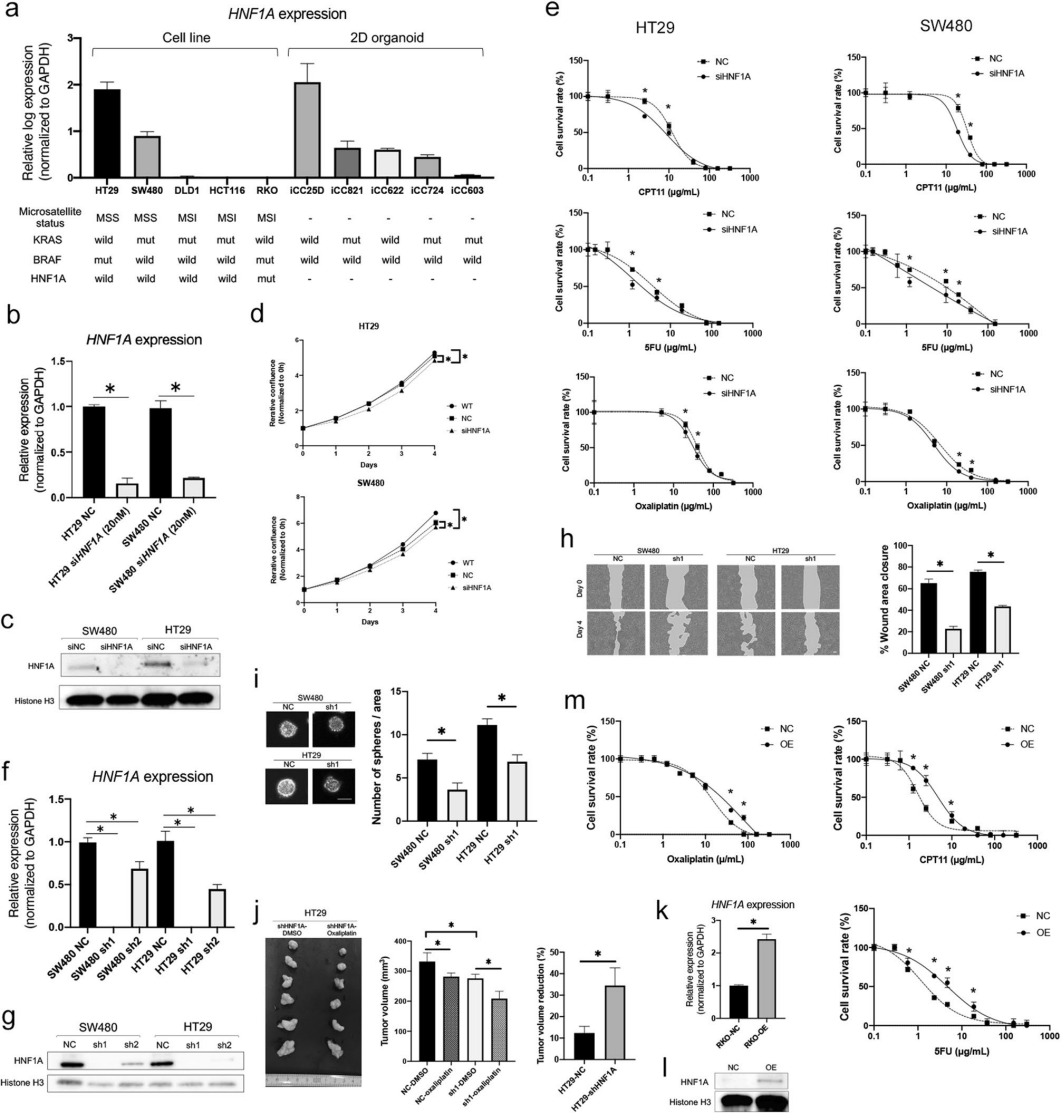
*HNF1A* mRNA expression in colorectal cancer (CRC) cells and inhibition of *HNF1A*. (**a**) *HNF1A* mRNA expression was evaluated in five CRC cell lines and five primary cultured cells (n = 3). Values are presented as means ± SEM. Microsatellite status, KRAS mutation status, BRAF mutation status, and HNF1A mutation status of each cell lines were shown. (**b**) The reduction in *HNF1A* expression was significant with HNF1A siRNA (siHNF1A) compared to levels in the siRNA negative control (NC) in two cell lines (n = 4). Values are presented as the means ± SEM (*P < 0.05, Wilcoxon’s rank sum test). (**c**) Western blotting of HNF1A and NC in siRNA negative control (NC) and si HNF1A in two cell lines. (**d**) Proliferation assay after siRNA-mediated inhibition in two CRC cell lines. There were significant differences between WT or NC and siHNF1A groups (n = 6). Values are presented as the means ± SEM (*P < 0.05, Wilcoxon’s rank sum test.). (**e**) Drug-sensitivity assay after siRNA treatment based on two CRC cell lines. Drug sensitivity was improved in the siHNF1A group compared to in the NC group (n = 4). Values are presented as the means ± SEM (*P < 0.05, Wilcoxon’s rank sum test). (**f**) Tow HNF1A-specific small hairpin RNAs (shRNAs) were transduced into SW480 and HT29. *HNF1A* mRNA expression was significantly decreased in these cells (n = 3). Values are presented as means ± SEM (*P < 0.05, Wilcoxon’s rank sum test). (**g**) Western blotting showed HNF1A expression was decreased in HNF1A shRNA (sh1, sh2) compared with the negative control (NC). (**h**) Representative images of wound healing assay and wound closure were inhibited in HNF1A shRNA (sh1) compared with the negative control (NC) (n = 4). Values are presented as the means ± SEM (*P < 0.05, Wilcoxon’s rank sum test). (**i**) Representative images of spheres and the number of spheres was reduced in HNF1A shRNA (sh1) compared to the negative control (NC) (n = 8). Values are presented as the means ± SEM (*P < 0.05, Wilcoxon’s rank sum test). (**j**) Representative image of xenograft tumors of HNF1A shRNA cells with/without oxaliplatin treatment and the tumor volume was reduced in HNF1A shRNA cells (sh1) compared with the negative control (NC). Oxaliplatin treatment further reduced the size of tumor, especially in sh1 (n = 6). Values are presented as the means ± SEM (*P < 0.05, Wilcoxon’s rank sum test). (**k**) *HNF1A* mRNA expression was elevated in the doxycycline-induced HNF1A overexpressed RKO cells (OE) compared with negative control without doxycycline (NC) (n = 3). Values are presented as the means ± SEM (*P < 0.05, Wilcoxon’s rank sum test). (**l**) Western blotting of HNF1A in HNF1A overexpressed RKO cells (OE) compared with the negative control (NC). (**m**) Drug-sensitivity assay in HNF1A overexpressed RKO cells (OE) compared with rhe negative control (NC). Drug sensitivity decreased in the OE group compared to in the NC group (n = 4). Values are presented as the means ± SEM (*P < 0.05, Wilcoxon’s rank sum test).

The mRNA expression of *HNF1A* was evaluated in five CRC cell lines and five 2DOs. Gene mutation status of KRAS, BRAF, HNF1A were searched from COSMIC (cancer.sanger.ac.uk)^23^ and Cancer Cell Line Encyclope^24^. The values of *HNF1A* expression, microsatellite status^25^, and gene mutation status were shown in Fig. 2a. Only RKO had HNF1A mutation, and *HNF1A* expression levels varied among the cells. HT29 and SW480 cells highly expressed *HNF1A*, which were microsatellite stable (MSS) tumors. The expression of HNF1A did not seem to be related to the status of KRAS and BRAF mutations. Next, HT29 and SW480 CRC cell lines were subjected to siRNA knockdown to assess the impact on cancer malignancy. A significant suppression of endogenous HNF1A by siRNA was confirmed using real-time RT–PCR (*P* < 0.05; Fig. 2b), and Western blotting (Fig. 2c, Supplementary Figure S7). Cell growth was then calculated to evaluate the proliferative properties of the cells, which revealed significant differences in cell numbers between the wild-type (WT; cells without transfection) or negative control (NC; cells with transfection of negative control) groups and the *HNF1A* siRNA (siHNF1A; cells with transfection of the siHNF1A) group for both CRC cell lines (*P* < 0.05; Fig. 2d). Next, anticancer drug sensitivity was evaluated in the NC and siHNF1A groups using, 5-fluorouracil (5-FU), CPT-11 (CPT11), and oxaliplatin, which are commonly used for mCRC chemotherapy^4^. The IC_50_s of each drug are summarized in Supplementary Table S5. Drug sensitivity was improved in the siHNF1A groups compared to in the NC group for both cell lines and all three drugs (*P* < 0.05; Fig. 2e). For further analysis, the HT29 and SW480 CRC cell lines were subjected to small hairpin RNA (shRNA) knockdown. Two *HNF1A*-specific shRNAs (sh1 and sh2) were transduced and HNF1A expression was decreased in both *HNF1A* shRNA (shHNF1A; cells with transfection of shHNF1A) compared to in the negative control (NC; cells with transfection of negative control) (Fig. 2f,g, Supplementary Figure S8). Cell migration and sphere formation were significantly inhibited by knockdown of *HNF1A* (Fig. 2h,i). Effects on cell proliferation and anti-cancer drug treatment were also examined in vivo using HT29 and oxaliplatin*.* Tumor growth was inhibited by knockdown of *HNF1A*, and anticancer drug treatment further reduced tumor progression (Fig. 2j). Knockdown of HNF1A increased tumor reduction by oxaliplatin. Furthermore, the effects of HNF1A were examined using RKO with doxycycline-induced Tet-On system. Doxycycline increased the expression of HNF1A (Fig. 2k,l, Supplementary Figure S9) and increased the IC50 level of the anticancer drug (Fig. 2m). From these results, it was notable that HNF1A strongly related anti-tumor drug sensitivity.

### The significance of HNF1A in drug resistance

**Figure 3:**
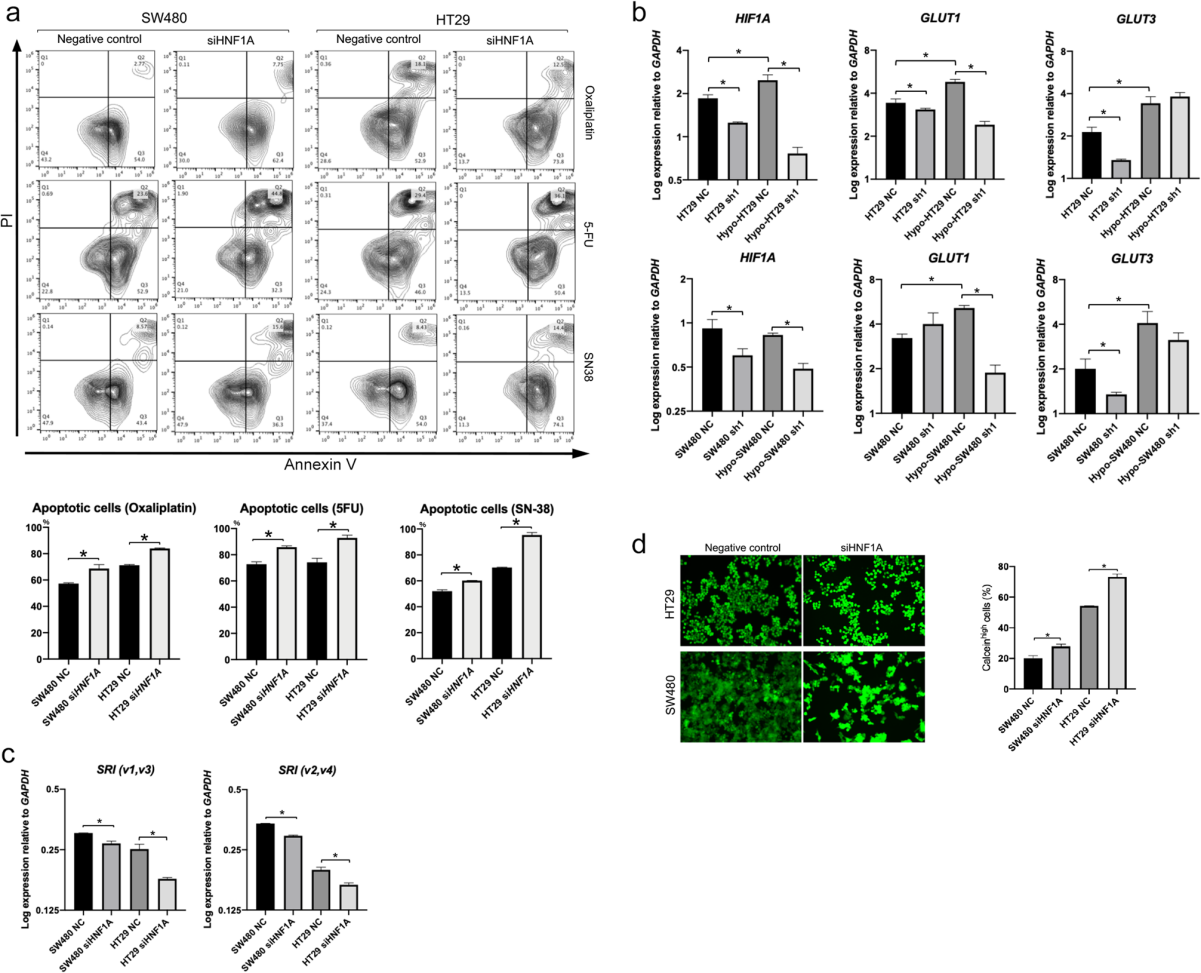
Analysis of downstream of HNF1A. (**a**) Flow cytometric analysis of apoptosis. siHNF1A significantly increased the number of annexin V-expressing cells in response to oxaliplatin, 5-FU, and CPT-11 in both cell lines (n = 3). Values are presented as means ± SEM (*P < 0.05, Wilcoxon’s rank sum test.). (**b**) *HIF1A*, *GLUT1*, and *GLUT3* expression was increased in hypoxia in HT29. *GLUT1* and *GLUT3* expression was increased in hypoxia (Hypo-) in SW480. There were significant differences in the expression of *HIF1A* and *GLUT1* between the negative control (NC) and siHNF1A groups in hypoxia in both cell lines. There were no significant differences in the expression of *GLUT3* in hypoxia in both cell lines (n = 4). Values are presented as the means ± SEM (*P < 0.05, Wilcoxon’s rank sum test.). (**c**) There were significant differences in the expression *SRI*, target gene of HNF1A, between negative control (NC) and siHNF1A groups (n = 4). Values are presented as the means ± SEM (*P < 0.05, Wilcoxon’s rank sum test.). (**d**) Representative images and flow cytometric analysis of multi-drug resistance assay. Calcein^high^ cells (> 1 × 10^5^) significantly increased in the siHNF1A group compared with that in the NC group (n = 3). Values are presented as the means ± SEM (*P < 0.05, Wilcoxon’s rank sum test.).

Knockdown of HNF1A significantly increased apoptotic cells in anti-tumor drug treatment (Fig. 3a). HNF1A is known to be deeply involved in glucose metabolism in normal tissues^13^. Glucose metabolism and anti-apoptosis are involved in treatment resistance, and hypoxia-inducible factor 1 subunit alpha (HIF1A) plays an important role^26^. In HT29, HIF1A was induced in hypoxia and suppressed by knockdown of *HNF1A* (*P* < 0.05; Fig. 3b). HIF1A is involved in inducing Glucose Transporter Type 1 *(GLUT1)* expression^27^ in hypoxia, and GLUT1 overexpression was reported to be associated with poor prognosis in various malignant tumors including CRC^28,29^. *GLUT1* was also induced in hypoxia and suppressed by knockdown of *HNF1A* (*P* < 0.05; Fig. 3b). Glucose Transporter Type 3 (*GLUT3)* was also examined, but no significant suppression was observed by knockdown of HNF1A. Furthermore, target genes of HNF1A as d transcription factor were predicted using Chip-Atlas^30^. Among the top 10 genes (Supplementary Table 6), we focused on Sorcin (SRI). HNF1A binding sites were identified shown in Supplementary Figure S10 using the Integrative Genomics Viewer^31,32^, and the expression of *SRI* was evaluated in NC and siHNF1A cells. SRI has four variant forms, and thus two primer sets were constructed; one was for variants 1 and 3 and the other was for variants 2 and 4. The expression of *SRI* was significantly lower in the siHNF1A group than in NC cells (Fig. 3c). SRI was reported to encode Sorcin, a calcium-binding protein that increases multidrug resistance (MDR) in cancer cells^33^. To evaluate the function of MDR proteins, an MDR assay was performed. Calcein-AM was rapidly excluded from cells expressing these proteins, and the remaining calcein-AM was degraded to calcein by elastase, generating calcein fluorescence. siHNF1A induced higher levels of calcein fluorescence than in the NC group (Fig. 3d). Thus, *HNF1A* knockdown suppressed MDR protein activity resulting in improved drug-sensitivity.

### miRNA targeting HNF1A improved drug sensitivity

**Figure 4:**
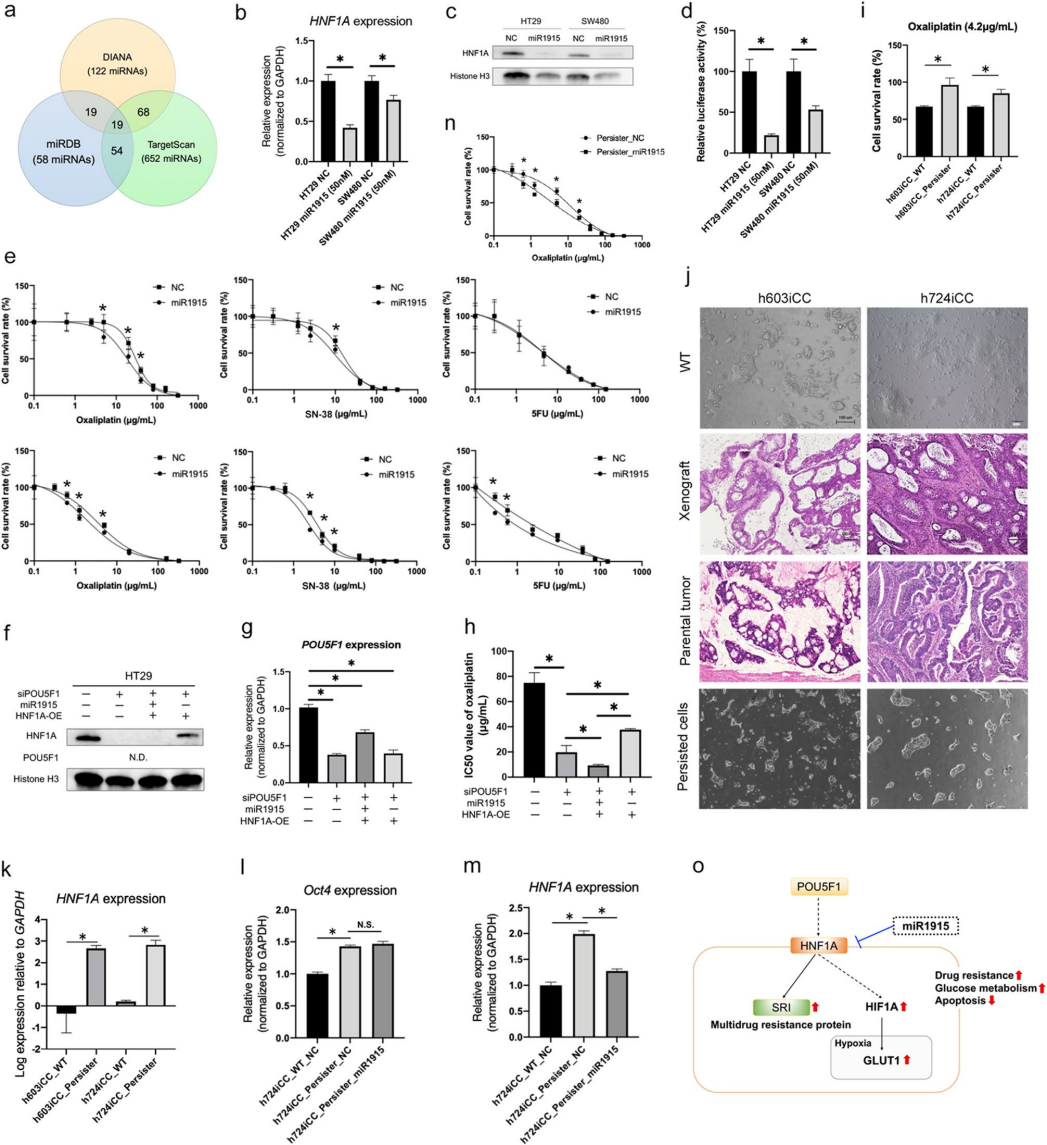
miRNA1915 inhibits HNF1A and improves drug sensitivity. (**a**) Overlapping genes among the three miRNA databases. (**b**) The reduction in *HNF1A* mRNA expression was significant with miR1915 transfected cells compared to in the miR negative control (NC) (n = 4). Values are presented as means ± SEM (*P < 0.05, Wilcoxon’s rank sum test). (**c**) Western blotting of HNF1A and POU5F1 in miR1915 transfected cells (miR1915) and the miR negative control (NC). (**d**) The activity of HNF1A observed as luciferase activity was suppressed by miR1915 (n = 5). Values are presented as the means ± SEM (*P < 0.05, Wilcoxon’s rank sum test). (**e**) Drug-sensitivity assay after miRNA inhibition in two colorectal cancer (CRC) cell lines. Sensitivity to oxaliplatin and CPT-11 was improved in the miRNA group compared to in the NC group (n = 4). Values are presented as the means ± SEM (*P < 0.05, Wilcoxon’s rank sum test). (**f**) Western blotting of rescue experiment under si*POU5F1* by overexpression of *HNF1A* and the effect of miR1915. *HNF1A* was suppressed by si*POU5F1* and re-expressed by doxycycline-induced overexpression (OE). miR1915 suppressed HNF1A in HNF1A-OE cells. POU5F1 was not detected. (**g**) The reductions in *POU5F1* expression were significant with *POU5F1* siRNA (si*POU5F1*) regardless of OE or miR1915 (n = 4). Values are presented as the means ± SEM (*P < 0.05, Wilcoxon’s rank sum test). (**h**) The IC50 value of oxaliplatin was decreased by si*POU5F1*, but increased by OE of HNf1A. Furthermore, it was suppressed again by miR1915 (n = 4). Values are presented as the means ± SEM (*P < 0.05, Wilcoxon’s rank sum test). (**i**) Cell survival rates were higher in drug-exposed persister cells (Persister) than in wild type (WT) cells (n = 4). Values are presented as means ± SEM (*P < 0.05). (**j**) Representative images of primary cultured cells. WT, two in vitro-cultured cell lines (h603iCC and h724iCC) from two patients; Xenograft, xenograft tumors from cultured cells were stained with hematoxylin and eosin; Parental tumor, surgically-resected specimens were stained with hematoxylin and eosin; Resistance, two in vitro-cultured cell lines were exposed to anti-cancer drugs for 4 weeks. Scale bars, 100 μm. (**k**) *HNF1A* mRNA expression was increased in drug-exposed persister cells (Persister) compared to in the WT group (n = 3). Values are presented as means ± SEM (*P < 0.05, Wilcoxon’s rank sum test). (**l**) *POU5F1* mRNA expression was increased in the drug-exposed persister cells (Persister) compared to in the wild type (WT) cells, but it was not decreased by miR1915. *HNF1A* mRNA expression was increased in Persister cells compared to in the WT cells, and it was decreased by miR1915 (n = 3). (**m**) *HNF1A* mRNA expression was increased in the drug-exposed persister cells (Persister) cells compared to the wild type (WT) cells (n = 3). Values are presented as the means ± SEM (*P < 0.05, Wilcoxon’s rank sum test). (**n**) Drug-sensitivity assay after miRNA inhibition in drug-exposed persister h724iCC cells. Sensitivity to oxaliplatin and CPT-11 was improved in the miRNA group compared to in the NC group (n = 4). Values are presented as means ± SEM (*P < 0.05, Wilcoxon’s rank sum test). (**o**) Simplified schema of the regulatory mechanisms discovered in this study relating to HNF1A and POU5F1.

Recent studies have revealed the potential of using miRNAs as therapeutic drugs^34^, and miRNAs targeting HNF1A were examined using three common miRNA databases, namely the DIANA-TarBase v.8^35^, miRDB^36^, and TargetScan Releases 7.2^37^. Overlapped 19 miRNAs were extracted among these databases (Fig. 4a), which were ranked in order according to their matched scores in each database (Supplementary Table S7). Among these miRNAs, we focused on the top-ranked has-miR-1915 (miR1915). The significant suppression of endogenous *HNF1A* expression by miR1915 was confirmed by real-time RT-PCR (*P* < 0.05; Fig. 4b) and Western blotting (Fig. 4c, Supplementary Figure S11). Reporter assay showed that miR1915 regulated *HNF1A* gene expression (Fig. 4d). Anti-cancer drug sensitivity was evaluated in the negative control (NC; cells with transfection of negative control) and the miR1915 (miR1915; cells with transfection of miR1915). Sensitivity to oxaliplatin and CPT-11 was improved in miR1915 compared with in the NC for both cell lines, and sensitivity to 5-FU was improved only in SW480 cells (Fig. 4e). Moreover, the interaction of *POU5F1, HNF1A,* and miR1915 was examined in more detail. Knockdown of *POU5F1* reduced *HNF1A* expression and improved drug sensitivity, but over expression of *HNF1A* reduced drug sensitivity (Fig. 4f–h, Supplementary Figure S12). In addition, miR1915 suppressed the expression of *HNF1A* and improved drug sensitivity. Finally, primary cultured 2DOs (h603iCC and h724iCC) were examined as clinical models. h603iCC indicated the form of mucinous adenocarcinoma in both xenograft tumor and parental tumor, and h724iCC indicated the form of tubular adenocarcinoma in both (Fig. 4j). They were representative pathological forms of colorectal cancer^5^. Oxaliplatin is a key drug for CRC^4^, and h603iCC and h724iCC were exposed to oxaliplatin (4.2 μg/mL) for 4 weeks. The colonies were slightly smaller in persister cells after exposure to oxaliplatin than before exposure (Fig. 4j). Persister cells showed higher viability than the original cells (WT; wild type cells unexposed to oxaliplatin) for the same concentration of oxaliplatin (Fig. 4i). *HNF1A* expression was significantly elevated in drug-exposed persister cells compared with the WT group (Fig. 4k). We examined the effect of miRNA1915 on these persister cells. *POU5F1* expression was also significantly elevated in drug-exposed persister cells compared with that in the WT group (*P* < 0.05), but there was no difference between the persister cells with negative control and with the miR1915 (Fig. 4l). However, *HNF1A* expression was significantly suppressed in the persister cells with miR1915 compared with the persister cells with negative control (Fig. 4m). Further, sensitivity to oxaliplatin was evaluated, and miRNA1915 also improved drug sensitivity in persister h724iCC cells (Fig. 4n). In summary, HNF1A is downstream of POU5F1 involving in drug resistance via anti-apoptosis and MDR protein activity, and miRNA1915 can be a therapeutic miRNA that suppresses HNF1A (Fig. 4o).

## Discussion

This study revealed that HNF1A is downstream of POU5F1 and high *HNF1A* expression in cancer tissue is an independent marker of poor prognosis, which is related to CRC recurrence and patient mortality. We could not confirm that the direct interaction of POU5F1 to HNF1A, but Chip-Atlas^30^ showed that HNF1A is a target gene of POU5F1 as a transcription factor in embryonic Stem Cells and HUES64. This is the first report indicating that *HNF1A* expression is an independent predictor of CRC prognosis after curative resection. Moreover, we revealed that HNF1A plays an important role in glucose metabolism of CRC by regulating HIF1A and regulated drug sensitivity via MDR protein.

It was previously reported that patients with CRC with HNF1A mutations have a better prognosis than those without mutations^17^, and thus HNF1A likely has a functional role in disease outcomes, particularly for patients with CRC administered chemotherapy. According to TCGA database, mutation of HNF1A was seen in 14 patients (2.2%) out of 594 patients^16^, the mutation type was missense in 6 patients and truncating in 8 patients. Therefore, we focused on the expression of HNF1A and examined the change in anticancer drug sensitivity by suppressing HNF1A. Furthermore, CRC was recently classified into four groups according to its molecular subtypes^38^. Patients with MSI tumors were reported to receive greater benefits from combination fluorouracil, leucovorin, and oxaliplatin adjuvant chemotherapy compared to those with MSS tumors^39^, and MSI-H tumors had more *HNF1A* mutations than MSS tumors^15^. In this study, those classified as MSS (HT29 and SW480) had higher *HNF1A* expression than those classified as MSI (DLD1, HCT116, and RKO) among the five cell lines. Thus, reported findings and our results suggest that HNF1A is a key gene in anticancer drug sensitivity in CRC. Biological assessments showed that *HNF1A* expression is related to tumor malignancy of CRC cells. An in vivo study showed that siRNA-mediated inhibition of *HNF1A* resulted in a significant reduction in cell growth and improved chemosensitivity. HNF1A dysfunction in normal cells causes abnormal fatty acid and glucose metabolism^12,13^ via involvement in GLUT1 and GLUT2 expression in pancreatic β-cells^40^. Therefore, we examined the relationship between HNF1A and glucose transporters, which regulated glucose metabolism in gastrointestinal tumors^29^. In addition, we also examined HIF1A, which is known as a key gene in glucose metabolism via GLUT1 and GLUT3, apoptosis, cell proliferation, and tumor angiogenesis, especially in hypoxia^26^. By the knockdown of *HNF1A*, *HIF1A* expression was suppressed, and *GLUT1* was suppressed, particularly in a hypoxic environment. GLUT1 was reported as a prognostic gene in CRC^29^, and our results suggest that HNF1A plays an important role in cancer glucose metabolism in association with HIF1A. However, the metabolism mechanisms are complex, so further studies are needed to show the importance and the relationship between these genes. Next, we focused on the role of HNF1A in chemosensitivity. It was reported that methylation in the HNF1A promoter regulates UGT1A1, which contributes to the metabolism of irinotecan^41^. In addition, we revealed that *HNF1A* knockdown improved chemosensitivity toward other two drugs, 5-FU and oxaliplatin. Chromatin immunoprecipitation (ChIP) analysis showed that HNF1A binds the SRI gene related to chemosensitivity as a transcription factor. SRI is overexpressed in many cancers^33^, including CRC^42^ and is considered as a useful marker of MDR. We revealed that knockdown of HNF1A suppressed *SRI* expression and MDR protein activity. Thus, HNF1A was thought to be involved in factors that are more widely related to anticancer drug resistance, and our results showed that HNF1A may be a therapeutic target for patients with CRC to suppress MDR protein activity, resulting in improvements in chemosensitivity. Finally, we examined the *HNF1A* expression in anticancer drug resistance using primary cultured 2DOs. 2DO is a model that can reproduce various characteristics of clinical tumors in gene expression, pathological features, and gene mutations^18^. Moreover, 2DO’s drug sensitivity reflected the clinical course of parental patients. We believe that it is important to verify the results not only cell lines but also 2DO as a clinical patient model. Recently, target therapy in CRC has been developed, and treatment outcomes have improved^4,43^. Many of the molecular target therapeutics molecules expressed on the cell membrane surface^44–48^, and it will take much time to develop drugs targeting nuclear transcription factors. However, the potential of using miRNAs as therapeutic drugs has been demonstrated^34^. Thus, we examined the therapeutic miRNAs targeting HNF1A for clinical applications. It was previously reported that miR1915 is downregulated in plasma samples from patients with chemoresistant CRC^49^. miR1915 extracted from the official database as possibly suppressing HNF1A reduced *HNF1A* mRNA expression and improved drug sensitivity in CRC cell lines. Further, exposure to anti-cancer agents increased *HNF1A* mRNA expression in primary cultured cells and suppression of *HNF1A* using miR1915 improved chemosensitivity. Our results suggest that in cases of high HNF1A expression or chemoresistant CRC after chemotherapy, inhibiting HNF1A can mitigate resistance to anti-cancer agents and augment chemotherapy. In addition, miR1915 can be a potential candidate for the suppression of HNF1A.

In conclusion, we identified a novel downstream target of POU5F1. HNF1A may be a useful prognostic indicator and therapeutic target for patients with CRC.

## Materials and methods

### Clinical tissue samples for HNF1A analysis

A total of 198 patients with CRC were registered and underwent resection of CRC and distant metastases at Osaka International Cancer Institute (OICI) from 2009 to 2013. No patients had administered chemotherapy or radiotherapy before surgery. For histological examination, resected specimens were fixed in formalin, processed through a graded ethanol series, embedded in paraffin, sectioned, and stained with hematoxylin and eosin and elastica van Gieson stain. Pieces of CRC specimens and adjacent normal colorectal mucosa were also frozen in liquid nitrogen immediately after resection and stored at − 80 °C until RNA extraction. After surgery, patients underwent follow-up blood and imaging examinations every 3–6 months according to Japanese guidelines^5^. Patients with stage III and IV lesions treated with R0 operations were administered adjuvant post-operative chemotherapy. Clinicopathological factors were assessed according to the tumor node metastasis (TNM) classification of the International Union Against Cancer^50^.

### Microarray analysis

Gene expression microarrays were analyzed for 2DOs, CRC tissue samples, normal colonic mucosal tissues, and CRC cell lines. Total RNA was prepared using an RNA Purification Kit (Qiagen, Hilden, Germany). A gene expression microarray (Agilent Technologies, Santa Clara, CA, USA) was constructed. Gene expression was analyzed using the Subio software platform (Subio, Inc., Kagoshima, Japan). The transcript profile was deposited with the accession number GSE99158. Other data generated or analyzed in this study are included in previous published article^18^.

### RNA preparation and expression analysis

Total RNA was prepared using an RNA Purification Kit (Qiagen). Reverse transcription was performed using a Transcriptor First Strand cDNA Synthesis Kit (Roche Diagnostics, Basel, Switzerland). Designed primers and corresponding universal probe libraries (Roche Diagnostics) and commercially available primers (Bio-Rad, Hercules, CA, USA) are listed in Supplementary Table S8. cDNA from the Human Reference Total RNA (Clontech, Mountain View, CA, USA) and RNA extracted from NTERA-2 cells were studied concurrently as positive controls. Quantitative assessment was performed by real-time reverse transcription-polymerase chain reaction (RT-PCR) using 100 nM universal probe libraries, a 0.1× FASTStart TaqMan Probe Master (Roche Diagnostics) for the designed primers, iTaq Universal SYBR Green Supermix (Bio-Rad) for commercially available primers, 100 nM primers, and 10 ng cDNA for cDNA amplification of target genes. PCR was performed with 20 µL of master mix in each well of a 96-well plate, and signals were detected with the CFX Connect Real-Time PCT Detection System (Bio-Rad). The thermocycler was programmed for 1cycle at 95 °C for 10 min, followed by 40 cycles at 94 °C for 10 s, 60 °C for 20 s, and 72 °C for 1 s.

### Immunohistochemistry

After deparaffinization and blocking, the sections were incubated with primary anti-HNF1A rabbit polyclonal antibody (ab204306; Abcam, Cambridge, UK) at a dilution of 1:200 overnight at 4 °C. The sections were incubated with biotinylated secondary antibody (Vectastain Universal Elite; Vector Laboratories, Burlingame, CA, USA) for 30 min, and signals were detected using the ImmPACT DAB kit (Vector Laboratories) with 5-min incubation. All sections were counterstained with hematoxylin.

### Culture of CRC cell lines

The human colorectal tumor cell lines HCT116, DLD‐1, and RKO cells, gifted by Dr. Bert Vongelstein (Johns Hopkins University, Baltimore, MD, USA), as well as SW480 (EC87092801, ECACC, UK) and HT29 (EC91072201, ECACC) cells were cultured in Dulbecco’s modified Eagle’s medium supplemented with 10% fetal bovine serum (Thermo Fisher Scientific, Waltham, MA, USA), 1% GlutaMAX‐I (Thermo Fisher Scientific), and 1% penicillin/streptomycin/amphotericin B (Wako Pure Chemical Industries, Osaka, Japan). The cells were incubated at 37 °C in a humidified atmosphere containing 5% CO_2_. Cells were harvested using 0.25% Trypsin–EDTA (Thermo Fisher Scientific, MA, USA) for further analysis.

### Primary culture of CRC cells

Primary cultured h603iCC and h724iCC cells (iCC25D, iCC821, iCC622, iCC724, iCC603, iCCh724, iCCh603) were established according to a previous report^18^ and cultured in 2 mL of modified stem cell culture medium^18^. The cells were incubated at 37 °C in a humidified atmosphere containing 5% CO_2_ and the medium was changed every 2–3 days. Cells were harvested using Accutase (Nacalai Tesque, Kyoto, Japan) for further analysis.

### Immunocytochemistry

Cultured cells were fixed with 4% formaldehyde and blocked. They were incubated with primary anti-HNF1A rabbit antibody (ab204306; Abcam, Cambridge, UK) at a dilution of 1:200 and anti-POU5F1 mouse antibody (#75463; Cell Signaling Technology, Beverly, MA) at a dilution of 1:200 overnight at 4 °C. Cells were incubated with secondary antibody (Goat anti-Rabbit IgG Secondary Antibody Alexa Fluor 488; A11008, Thermo Fisher Scientific, Waltham, MA, USA, Goat anti-Mouse IgG Secondary Antibody Alexa Fluor 594; A11005, Thermo Fisher Scientific) at a dilution of 1:2000 for 90 min. The side was mounted in Prolong Gold with DAPI (Thermo Fisher Scientific) overnight.

### DNA mutation analysis for 2DOs

DNA was extracted from 2DOs using a DNA Purification Kit (Qiagen) and analyzed for the presence of *KRAS* and *BRAF* mutations using a BNA Real-time PCR Mutation Detection Kit Extended RAS (Riken Genesis, Tokyo, Japan). Sanger sequencing was performed to confirm the mutations.

### Small interfering RNA inhibition of cultured cells

siRNA (Silencer Select Pre-designed siRNA; HNF1A Cat. no. 4392420, Validated Stealth RNAi; POU5F1 Cat. No. HSS143403, Thermo Fisher Scientific), and negative control siRNAs (Silencer Select Negative Control no. 1 siRNA Cat. no. 4390843, Stealth RNAi Negative Control, Medium GC Duplex Cat.no. 12935-112, Thermo Fisher Scientific) were used. 2DOs and CRC cell lines were transfected with siRNA at a final concentration of 20 nM for HNF1A and 10 nM for POU5F1 using Lipofectamine RNAiMAX (Thermo Fisher Scientific) according to the manufacture’s protocol. Cells were incubated in glucose-free Opti-MEM (Fisher Scientific), and used for further analysis.

### shRNA transduction of cultured cells

HNF1A-specific small hairpin RNAs in the pLKO puromycin-resistant vector (MISSION shRNA TRCN0000017195 (described as sh1) and TRCN0000017196 (described as sh2)) were purchased from Sigma (SIGMA Mission shRNA, St. Louis, MO, USA). Trans-IT-293 Transfection Reagent (TAKARA Bio) was used to transfect 293T cells to create a lentivirus constitutively expressing the shHNF1A sequence. After 48 h, the lentivirus supernatant was collected, and the purified supernatant was used to infect CRC cells at 50% confluence. The proper multiplicity of infection of lentivirus was added to the cells and incubated for 96 h. The medium was changed to fresh medium containing 2 µg/mL puromycin. Knockdown of HNF1A was confirmed by RT-PCR and western blotting before further analysis.

### Western blotting

Cells were collected and lysed with RIPA lysis buffer, and lysates were centrifuged at 10,000 × g for 20 min. Protein concentration of collected supernatants was measured by Quick Start Bradford Protein Assay (Bio-Rad). Equal 10 μg proteins were loaded into 10% TGX polyacrylamide gels (Bio-Rad). The proteins were electrophoresed at 200 V for 40 min and transferred to Poly Vinylidene Di-Fluoride membranes. Immunoblots were detected using iBind Flex Western Device (Thermo Fisher Scientific Inc.) according to the manufacturer’s instructions. Anti-HNF1A rabbit polyclonal antibody (ab204306; Abcam) at a dilution of 1:150 and anti-Histone H3 rabbit monoclonal antibody (#4499, Cell Signaling Technology) at a dilution of 1:2000 were used as primary antibodies. The iBind Western System was run for 3 h with HRP-linked secondary antibody (#7074, Cell Signaling Technology) at a dilution of 1:2000. Protein expressions were visualized by Luminescent Image Analyzer LAS-3000 (Fujifilm Corporation, Tokyo, Japan). All experiments were independently performed in triplicate and a representative figure was shown.

### In vitro cell proliferation assays

1 × 10e^5^ cells of *HNF1A* knockdown cells (si), negative control cells (NC), and wild-type cells (WT) were seeded into 12-well plates. Proliferation was evaluated over time in the same well by live cell imaging using IncuCyte S3 Live-Cell Analysis System (Sartorius, USA) on adhesion cell fluence. Values are presented as mean ± SEM from independent experiments performed six times.

### In vitro drug-sensitivity assays

After incubating the transfected cells overnight, the cells (1 × 10^4^ per well for 2DOs and 5 × 10^3^ per well for cell lines) were added to 96-well plates and incubated for 48 h. The cells were exposed to oxaliplatin (22600 AMX0983, Nippon Kayaku Co., Ltd., Saitama, Japan), camptothecin (017-13424, Wako Pure Chemical Industries) as CPT-11, and 5-FU (068-01401, Wako Pure Chemical Industries) for 96 h. The percentage of viable cells was determined using a cell counting kit solution (CCK-8; Dojindo Molecular Technologies, Kumamoto, Japan) compared to the number of cells without drug. Values are presented as the mean ± SEM from independent experiments performed four times.

### Sphere formation assay

Cells (1 × 10e^5^ per well) were seeded into 6-well low-attachment plates (#3471; Corning Incorporated, NY, USA) and cultured in 2 mL of serum-free culture medium containing 10 ng/mL bFGF (064-05381; Wako Pure Chemical Industries) and 1 ng/mL EGF (PMG8043; Thermo Fisher Scientific). They were cultured for three weeks, and the number of spheres with a diameter of 50 μm or more per field of view was measured at a 100× magnification microscope (Keyence, Itasca, IL, USA).

### Wound healing assay

Cells (3 × 10e^4^ per well) were seeded into 96-well plates. Scratches were made with a 200 μL chip, and cell migration was observed by live-cell imaging in IncuCyte (Essen Bioscience, Ann Arbor, MI, USA). Closed wound area after 4 days was calculated by area.

### Assays in hypoxia

Cells (1 × 10e^5^ per well) were seeded into 12-well plates. After overnight incubation, the cells were cultured under 5% hypoxia conditions using BIONIX-2 (Sugiyama-Gen Co., Ltd., Tokyo, Japan) for 48 h. The cells were harvested for further analysis.

### Apoptosis assay based on flow cytometry

On the day after transfection, siHNF1A and NC cells (5 × 10^5^ per well) were added to 6-well plates, and 24 h later exposed to oxaliplatin (5 μg/mL), camptothecin (2.5 μg/mL) as CPT-11, and 5-FU (1 μg/mL). CRC cell lines were then incubated for 24 h, then harvested with Accutase (Thermo Fisher Scientific). Cells were treated with annexin V (A35110, Thermo Fisher Scientific) and propidium iodide solution (130-093-233, Miltenyi Biotec, Bergisch Gladbach, Germany) according to the manufacturer’s protocols. The relative fluorescence intensities were measured using an SH800 cell sorter (SONY, Tokyo, Japan). Data were analyzed with FlowJo 10.2 software (FlowJo LLC, Ashland, OR, USA). Independent experiments were performed three times.

### Multi-drug resistance (MDR) assay

On the day after transfection, siHNF1A and NC cells (1 × 10^5^ per well) were added to 12-well plates. After overnight incubation, Calcein AM Solution (Multi Drug Resistance assay kit (600370), Cayman Chemical, Ann Arbor, MI, USA) was added to each sample well and incubated for 30 min according to the manufacturer’s protocol. The cells were harvested using Accutase (Thermo Fisher Scientific), and propidium iodide solution (Miltenyi Biotec) was used to stain dead cells. The samples were analyzed with an SH800 cell sorter (SONY), and data were analyzed with FlowJo 10.2 software (FlowJo LLC). Independent experiments were performed three times.

### miRNA inhibition in cultured cells

CRC cell lines (HT29 and SW48), 2DO (h724iCC), hsa-miR-1915 miRNA (AccuTarget hsa-miR-1915 miRNA mimic miRBase ver.21, Bioneer, Daejeon, Korea), and negative control miRNA (AccuTarget miRNA inhibitor Negative control #1, Bioneer) were used. Several concentrations were examined, and finally CRC cells were transfected with miRNA at a concentration of 50 nM using Lipofectamine RNAiMAX according to the manufacture’s protocol. Cells were incubated in glucose-free Opti-MEM (Thermo Fisher Scientific), and used for further analysis.

### Overexpression of HNF1A in cultured cells

CRC cell lines (RKO and HT29) were used. FR_HNF1A plasmid gifted from Gerhart Ryffel (Addgene plasmid # 31104; http://n2t.net/addgene:31104; RRID:Addgene_31104)) was transfected by Neon Transfection System (Thermo Fisher Scientific) at a 5 μg per well according to the manufacture’s protocol. 2 ug/mL doxycycline (Clontech, Palo Alto, CA) was used for over expression.

### Xenograft model for histological examination of primary cultured cells

Histological examination of primary cultured cells (2DOs) was performed using a xenograft model. Accutase-dissociated cells (5 × 10^5^ cells) suspended in Matrigel (BD Biosciences, Franklin Lakes, NJ, USA) were subcutaneously transplanted into the dorsal flanks of 7-week-old male non-obese diabetic/severe combined immunodeficiency mice (CLEA, Tokyo, Japan). Each 2DOs was injected into different mice. Mice were sacrificed when the tumors reached a diameter of 10 mm. Mice were weighed weekly and no mouse reduced body weight. Xenograft tumors were fixed in formalin, processed through a series of graded concentrations of ethanol, embedded in paraffin, and sectioned. Sections were stained with hematoxylin and eosin (H&E).

### Evaluation of the proliferative capacity of HNF1A knockdown cells using a flank xenograft model

HT29 cells (shHNF1A and NC cells, 2 × 10^6^ cells per site) suspended in Matrigel (BD Biosciences, Franklin Lakes, NJ, USA) were subcutaneously transplanted into the dorsal flanks of 7-week-old male non-obese diabetic/severe combined immunodeficiency mice (CLEA, Tokyo, Japan). After 3 days, all tumors were confirmed, and the mice were randomly allocated to the control group (DMSO) or oxaliplatin group. Oxaliplatin (Nippon Kayaku Co.) was given by SC injection at 6 mg/kg twice a week. After three weeks, all tumors were resected and measured.

### Statistics

*HNF1A* expression levels in CRC and normal colorectal mucosa, and the relationships between *HNF1A* expression levels and clinicopathological factors were analyzed using Wilcoxon’s rank sum test, χ^2^ test, and Tukey’s honestly significant difference test. Kaplan–Meier survival curves were plotted and compared using the generalized log-rank test. Prognostic factors were identified by univariate and multivariate analyses using a Cox proportional hazards regression model. In vitro assay results were analyzed using the Wilcoxon’s rank test. All test results were analyzed with JMP software (ver. 11.2; SAS Institute, Cary, NC, USA). A *P* value < 0.05 was considered statistically significant. Gene network was visualized with nodes and edges^51^ using each gene expression data of microarray. It was constructed by R 3.1.3 (CRAN; the R Foundation for Statistical Computing, Vienna, Austria). Edges showed the correlation between genes, and the correlation coefficient was indicated by the thickness of the line. Genes having a coefficient of 0.3 or more were connected by a line, and nodes showed the centrality. The used main packages were tidyr (maintained by Hadley Wickham), ggraph (maintained by Thomas Lin Pederson), and tidygraph (maintained by Thomas Lin Pedersen).

### Study approval

OICI Review Board and OICI Animal Research Committee approved this study and written informed consent for the study was obtained from all participants according to the ethics guidelines of the OICI. All experimental protocols, including humans and animals were in accordance with the guidelines of the OICI and Declaration of Helsinki. This study was carried out in compliance with the ARRIVE guidelines.

## Supplementary information

Supplementary Informations.
