# Supplementary material for: HNF1A regulates colorectal cancer progression and drug resistance as a downstream of POU5F1
Source: Sci Rep. 2021 May 14;11:10363. doi: 10.1038/s41598-021-89126-2 (PMC8121855; doi:10.1038/s41598-021-89126-2)
Supplement: Supplementary file 1 — Supplementary Informations. [file 41598_2021_89126_MOESM1_ESM.pdf]

HNF1A* regulates colorectal cancer progression and drug resistance as a downstream of *POU5F1

Shiki Fujino, MD, PhD[1,2], Norikatsu Miyoshi, MD, PhD[1,2*], Aya Ito[1], Masayoshi Yasui, MD, PhD[3], Chu Matsuda, MD, PhD[3], Masayuki Ohue, MD, PhD[3], Mamoru Uemura MD, PhD[1], Tsunekazu Mizushima, MD, PhD[1], Yuichiro Doki, MD, PhD[1] , Hidetoshi Eguchi, MD, PhD[1]

[1] Department of Gastroenterological Surgery, Osaka University Graduate School of Medicine, 2-2, Yamadaoka, Suita-City, Osaka 565-0871, Japan Innovative Oncology Research and Translational Medicine, Osaka International Cancer Institute, 3-1-69, Otemae, Chuo-ku, Osaka 541-8567, Japan

[2] Innovative Oncology Research and Translational Medicine, Osaka International Cancer Institute, 3-1-69, Otemae, Chuo-ku, Osaka 541-8567, Japan

[3]Department of Surgery, Osaka International Cancer Institute, 3-1-69, Otemae, Chuo-ku, Osaka 541-8567, Japan

***Correspondence to**: Norikatsu Miyoshi, MD, PhD, FICS, FACS, Department of Gastroenterological Surgery, Osaka University Graduate School of Medicine, 2-2, Yamadaoka, Suita-City, Osaka, 565-0871, Japan.

E-mail: nmiyoshi@gesurg.med.osaka-u.ac.jp; Tel: +81-(0)6-6879-3251; Fax: +81-(0)6-6879-3259

Supplementary materials

Supplementary Figure S1. FACS analysis of 603iCC (2DO)

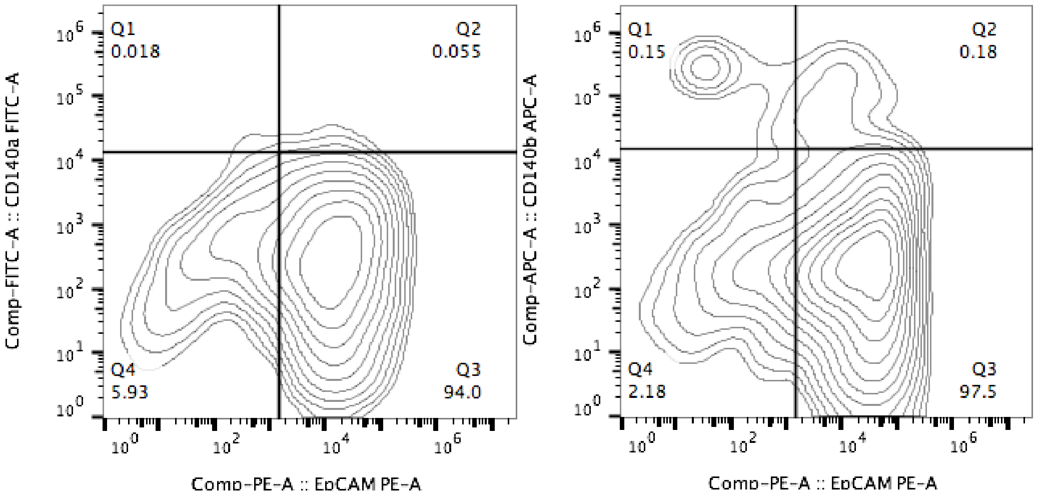

Most cells (93-99%) expressed epithelial cell adhesion molecule (EpCAM). There were few cells expressing only 140a and 140b as a stromal marker. (n=3)

Supplementary Figure S2. Volcano plots and the hierarchical clustering analysis of extracted 538 probes

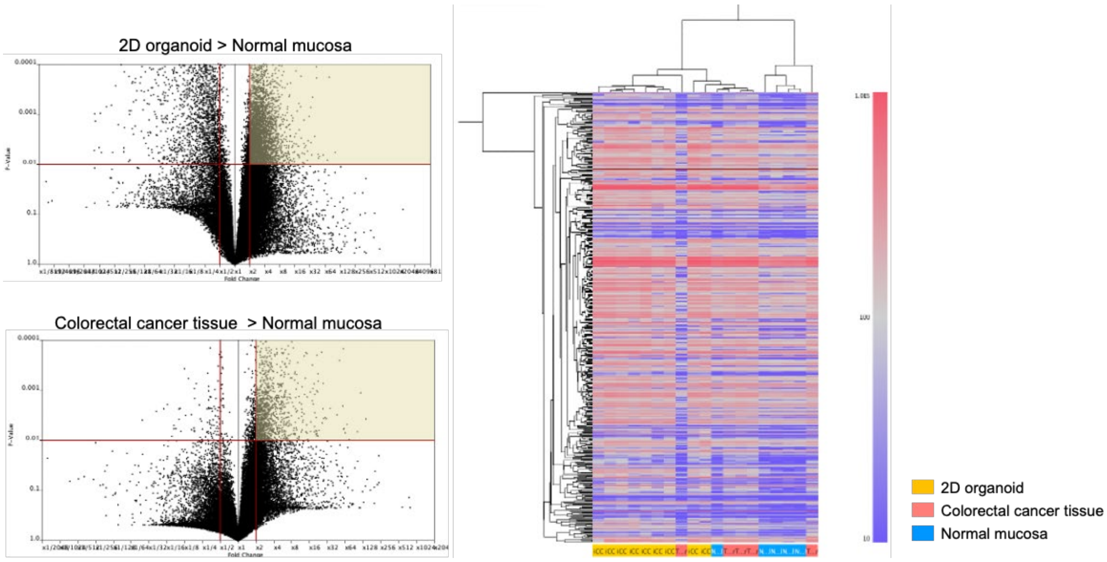

Supplementary Figure S3. Prognosis based on TCGA database

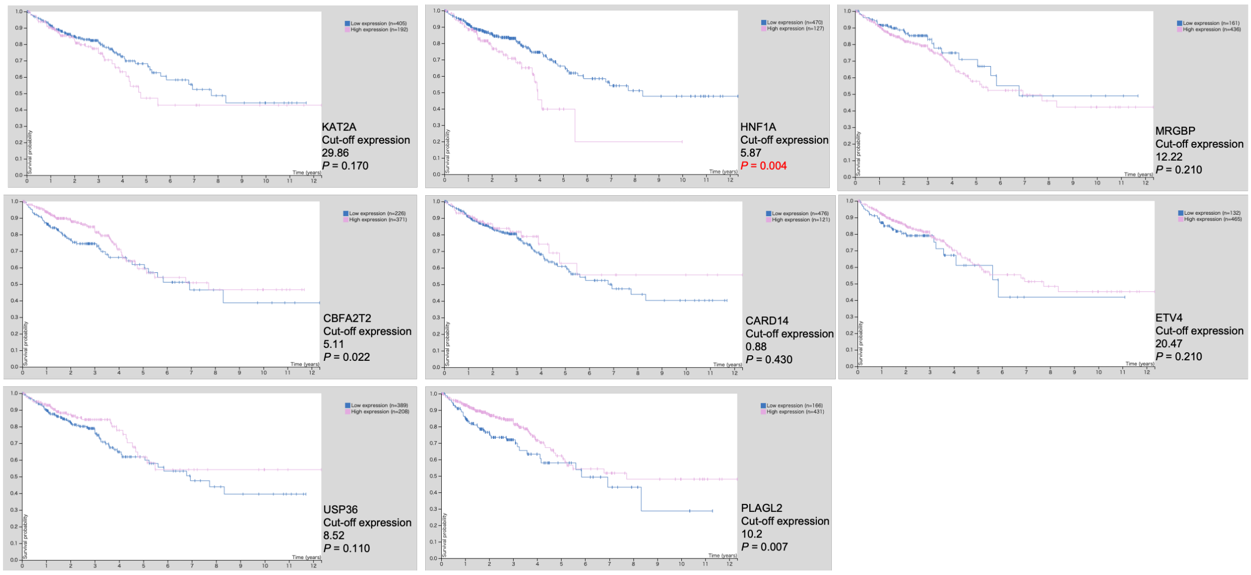

An analysis of the TCGA database showed that overall survival was lower in patients with high *HNF1A* mRNA expression levels (n =126) compared with that in individuals with low expression levels (n = 471) (P = 0.008, log-rank test). Low expression of *CBFA1T2* (n=226) was correlated with poor prognosis compared with high expression (n=372) (P = 0.022, log-rank test). Low expression of *PLAGL2* (n=166) was correlated with poor prognosis compared with high expression (n=431) (P = 0.007, log-rank test). There was no significance in other five genes.

Supplementary Figure S4. Western blot analysis of full-length gels and blots of Figure 1f

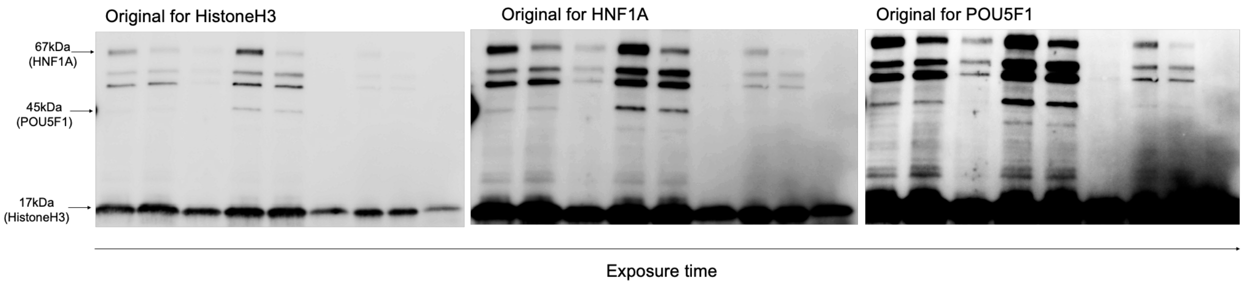

Supplementary Figure S5. *NANOG* expression in siPOU5F1 primary cultured cells

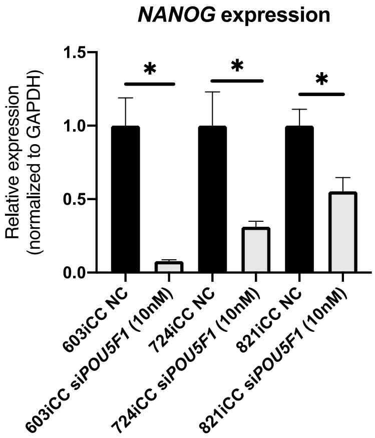

The reductions in *NANOG* expression were significant with *POU5F1* siRNA (si*POU5F1*) compared to levels in the siRNA negative control (NC) groups in all three 2DOs. (n = 4) Values are presented as means ± SEM (* $P < 0.05$, Wilcoxon's rank sum test)

Supplementary Figure S6. Immunohistochemistry of HNF1A in colon and small intestine

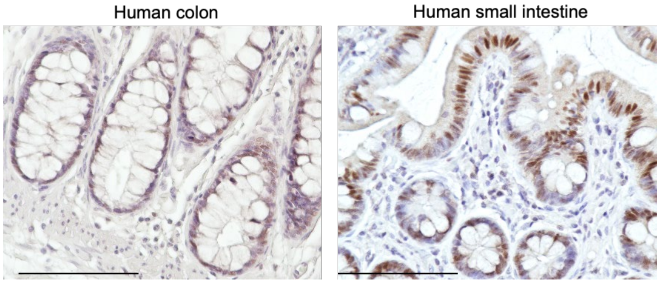

Scale bars, 100 μm

Supplementary Figure S7. Western blot analysis of full-length gels and blots of Figure 3a

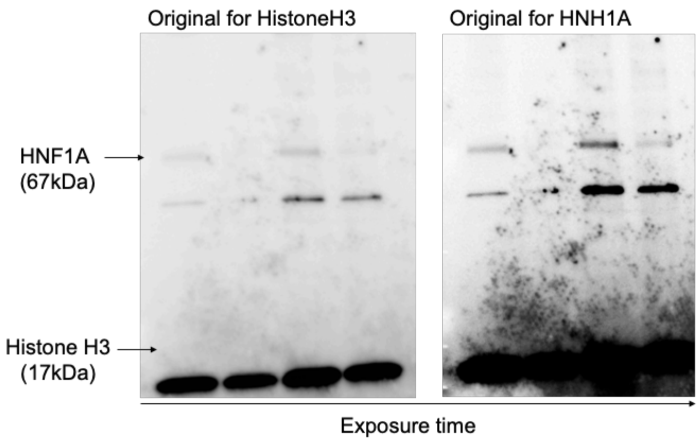

Supplementary Figure S8. Western blot analysis of full-length gels and blots of Figure 2g

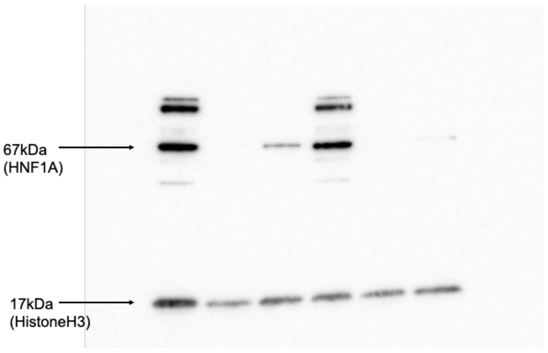

Supplementary Figure S9. Western blot analysis of full-length gels and blots of Figure 2l

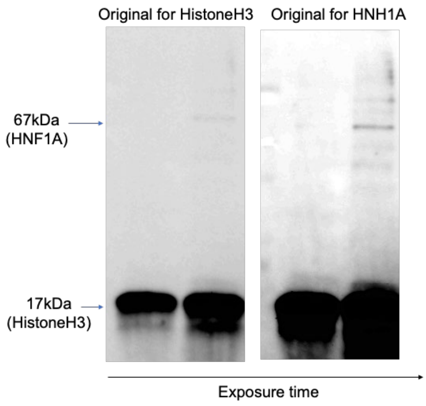

Supplementary Figure S10. *T*ranscription factor binding sites for HNF1A in Sorcin DNA sequences.

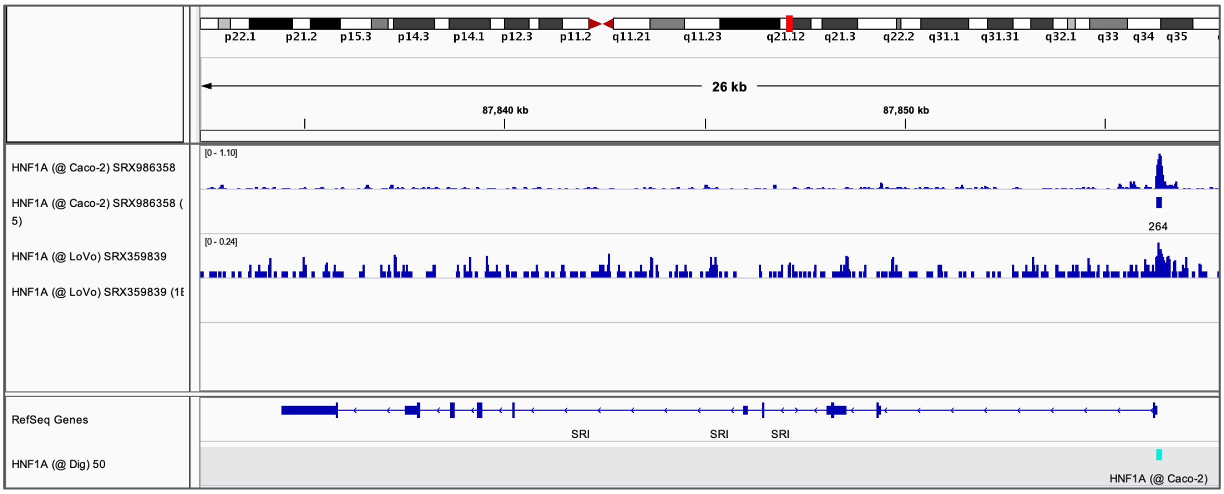

Supplementary Figure S11. Western blot analysis of full-length gels and blots of Figure 4c

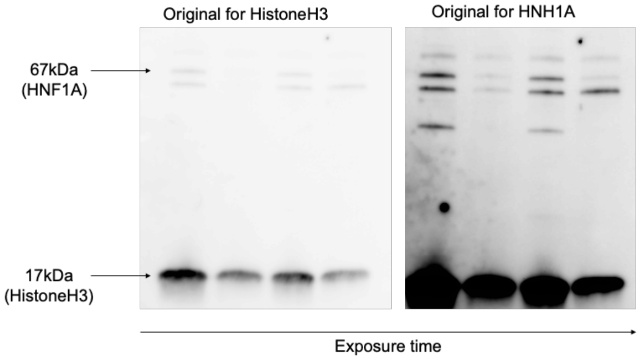

Supplementary Figure S12. Western blot analysis of full-length gels and blots of Figure 4f

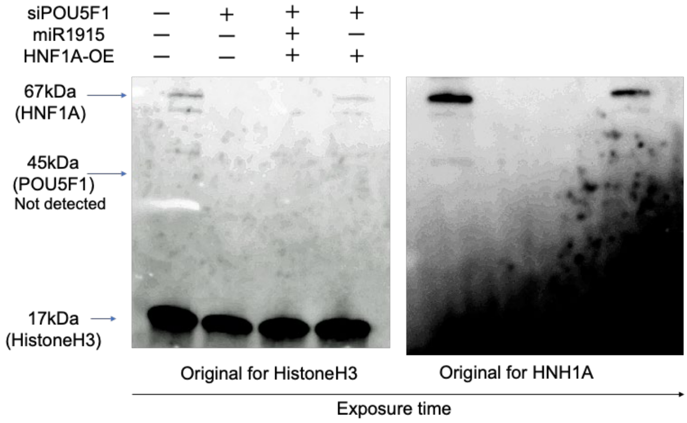

Supplementary Table S1. Extracted genes from microarray data set

GENE_SYMBOL	Fold Change (CRC tisssue/Normal mucosa)	P-Value (CRC tisssue/Normal mucosa)	Fold Change (2DO/Normal mucosa)	P-Value (2DO/Normal mucosa)
ACTR5	2.6007242	0.002702499	3.2216089	3.29E-05
ANKRD13B	5.10646	0.006340602	5.037356	0.005162392
ASCL2	18.047272	7.71E-05	12.625852	3.55E-04
ATAD3B	2.3563848	0.001124741	5.0208063	1.94E-06
BCL11B	5.6352196	1.07E-05	3.7484183	0.002325877
BRAT1	2.2705905	2.37E-04	2.767444	3.00E-04
C15orf62	5.8624363	3.40E-04	17.499067	1.20E-05
CARD14	5.6159167	0.001030086	5.7345033	5.06E-04
CBFA2T2	2.7488673	0.004288329	3.4741125	1.76E-04
CD3EAP	2.8345692	0.001983517	4.5889435	8.37E-04
CEP164	2.0949519	1.58E-04	2.2090287	0.00536012
CEP250	2.4755936	0.009819262	3.000186	0.00168374
CHTF18	2.8745975	9.48E-04	4.918887	2.06E-04
CLCN7	2.5373287	1.13E-04	4.8489666	2.86E-06
CLUHP3	2.4516058	0.003222721	3.1457863	8.29E-05
CPNE7	38.327812	5.93E-05	6.817908	0.004561269
DDX56	2.0121477	3.93E-04	3.318339	1.10E-05
DGKZ	2.1145642	0.007079882	3.5554516	3.16E-05
DHX34	2.414038	0.004426001	3.3116899	3.41E-05
EIF3B	2.0023036	9.56E-04	3.4580317	1.84E-04
ETV4	27.668062	2.95E-04	50.701252	1.67E-09
GPC2	2.093818	0.006330951	3.3606143	0.001884945
GTF2IRD1	6.6503496	2.39E-05	14.820366	3.87E-08
HNF1A	2.2075078	6.37E-04	5.028904	3.85E-10
KAT2A	2.3386452	0.005910468	2.8982682	0.001219246
KCNJ14	2.8382833	0.001554828	6.0285487	1.82E-04
KLHL31	4.829533	0.009580796	11.508928	3.97E-04
LTB4R	2.0760553	4.13E-05	3.5700784	2.96E-05
MRGBP	3.8491638	1.77E-04	7.6792717	4.01E-07
MYBL2	3.365287	0.006952426	5.515222	0.003147385
NPEPL1	2.3956563	0.008784014	2.895236	7.98E-04
NSUN5	2.2134104	0.001171444	3.9940946	2.68E-07
OGFR	2.1721795	0.001294366	2.1965659	0.001609913
PBX4	3.655537	0.005184078	6.178707	7.14E-04

PCP2	3.273973	0.006895416	2.6210136	2.92E-04
PLAGL2	2.424846	0.004299475	3.633566	7.82E-05
RAE1	2.6787562	0.001006118	3.5519028	2.48E-05
RHPN1	9.524554	0.003904573	8.278585	0.001046837
RHPN1-AS1	11.234191	0.00107761	7.771614	0.00280549
RNF43	5.297531	0.004413701	6.4659357	6.05E-05
SLC7A6	2.124334	0.006213036	3.7031841	1.15E-05
SNHG17	3.238493	0.001960859	4.2119837	2.44E-04
SPACA4	2.8980696	0.005852006	5.463606	4.45E-04
SPATA33	2.7168217	0.007852118	6.0080376	1.37E-05
TGIF2	6.1790075	6.28E-04	4.6817656	0.001278674
TONSL	3.922438	0.001746248	9.080728	6.73E-07
TOP1MT	4.8998075	6.88E-04	3.8754423	4.24E-04
USP36	2.311962	0.001725685	3.12476	1.60E-04
VEGFA	4.477089	0.00183693	5.371283	1.81E-04
YTHDF1	2.013948	0.002972835	2.8509667	2.59E-05
ZNF598	2.6806543	0.002944175	4.287579	6.75E-07

Supplementary Table S2. Enriched gene ontology terms

Term	Tumor biology	P-value
blastocyst development	Yes	4.50E-02
placenta development		9.00E-02
positive regulation of protein phosphorylation	Yes	4.50E-02
chromatin remodeling	Yes	2.20E-02
regulation of transcription from RNA polymerase II promoter	Yes	3.00E-02
transcription from RNA polymerase II promoter	Yes	4.00E-04
histone acetylation	Yes	8.70E-02
post-embryonic camera-type eye development		1.10E-02
positive regulation of transcription from RNA polymerase II promoter	Yes	1.50E-02
regulation of mitophagy	Yes	8.50E-02
positive regulation of protein targeting to mitochondrion	Yes	2.20E-02

Supplementary Table S3. Genes in enriched gene ontology terms relating tumor biology

HNF1 homeobox A (HNF1A)
lysine acetyltransferase 2A (KAT2A)
PLAG1 like zinc finger 2 (PLAGL2)
vascular endothelial growth factor A (VEGFA)
B-cell CLL/lymphoma 11B (BCL11B)
ETS variant 4 (ETV4)
MRG domain binding protein (MRGBP)
MYB proto-oncogene like 2 (MYBL2)
ubiquitin specific peptidase 36 (USP36)
BRCA1 associated ATM activator 1 (BRAT1)
caspase recruitment domain family member 14 (CARD14)
POU class 5 homeobox 1 (POU5F1)
CBFA2/RUNX1 translocation partner 2 (CBFA2T2)
GTF2I repeat domain containing 1 (GTF2IRD1)
PBX homeobox 4 (PBX4)
topoisomerase (DNA) I, mitochondrial (TOP1MT)

Supplementary Table S4. Patient's characteristics

**Factors**	**N=198**
Gender (Male / Female)	110 / 88
Age*, year	66 (16-88)
CEA*, ng/mL	4.2 (0.5-2171.0)
Histological grade (tub1/ tub2 / muc / por)	41 / 147 / 4 / 6
Tumor invasion (T2 / T3 / T4)	17 / 94 / 87
Lymph node metastasis (N0 / N1 / N2)	78 / 64 / 56
Lymphatic invasion (Absent / Present)	83 / 115
Vascular invasion (Absent / Present)	44 / 154
Stage (I / II / III / IV)	10 / 66 / 82 / 40
Surgical resection (R0 / R1-2)	173 / 25

*Continuous variable

Underlined values indicate P < 0.05.

tub1: well-differentiated adenocarcinoma, tub2: moderately differentiated adenocarcinoma, muc: mucinous adenocarcinoma, por: poorly differentiated adenocarcinoma.

Supplementary Table S5. IC 50 of anti-cancer drugs

	HT29 NC	HT29 siHNF1A	SW480 NC	SW480 siHNF1A
5FU (µg/mL)	3.5	1.4	27.2	4.0
SN-38 (CPT11) (µg/mL)	12.3	8.4	33.6	19.1
Oxaliplatin (µg/mL)	39.5	30.4	6.4	4.8

Supplementary Table S6. Target genes of *HNF1A* predicted by Chip-Atlas

Target genes	HNF1A Average	SRX986358_Caco-2
MTRNR2L2	321.8	609
RGPD2	279.8	607
MTRNR2L8	306.2	568
SPINK1	310.8	429
DDC	248.2	421
MTRNR2L9	133.6	308
SGK2	324.8	269
SRI	342.2	264
SRC	100.2	225
GPR39	429	215

Supplementary Table S7. miRNAs targeting HNF1A predicted by databases

miRNA name	Total score	Ranking
hsa-miR-1915-3p	6	1
hsa-miR-6764-5p	7	2
hsa-miR-548at-5p	10	3
hsa-miR-484	17	4
hsa-miR-6748-3p	21	5
hsa-miR-3155b	22	6
hsa-miR-3155a	23	7
hsa-miR-4690-5p	27	8
hsa-miR-4438	32	9
hsa-miR-1343-3p	35	10
hsa-miR-1205	35	11
hsa-miR-604	35	12
hsa-miR-6721-5p	35	13
hsa-miR-6783-3p	36	14
hsa-miR-6787-3p	39	15
hsa-miR-6815-3p	42	16
hsa-miR-4650-5p	42	17
hsa-miR-6852-5p	52	18
hsa-miR-4755-3p	54	19

Table S8. **Primer lists**

Primer	Sequence 5′–3′	UPL No.
HNF1A-L	GAGGCAGAAGAACCCTAGCA	25
HNF1A-R	GACACCCCTCTCTGGATGC	
GAPDH-L	AGCCACATCGCTCAGACAC	60
GAPDH-R	GCCCAATACGACCAAATCC	
POU5F1-L	GCTTCAAGAACATGTGTAAGCTG	69
POU5F1-R	CACGAGGGTTTCTGCTTTG	
GLUT1_L	CCAGCAGCAAGAAGCTGAC	52
GLUT1_R	TGATGACTCCAGTGTTGTAGCC	
GLUT3_L	CCGCTGCTACTGGGTTTTAC	4
GLUT3_R	CTTTCAGGGCAAAATGGAAG	
SRIv1v3_L	AGTCTGCAGCATGGCGTA	70
SRIv1v3_R	GCGGATCCTGAGTTTGTCC	
SRIv2v4_L	CGGCTTATGGTTTCAATGCT	26
SRIv2v4_R	TGTTGTCTCCAGCCATTCAG	

Commercially available primers:

HIF1A (PrimePCR SYBR Assay, Desalt 200R Wet-Validated, Predesign. 10025636) (Bio-Rad, Hercules, CA, USA)
